# Transvenous lead extraction in children with bidirectional rotational dissection sheaths

**DOI:** 10.3389/fcvm.2023.1256752

**Published:** 2023-09-08

**Authors:** Roland Heck, Björn Peters, Pia Lanmüller, Joachim Photiadis, Felix Berger, Volkmar Falk, Christoph Starck, Peter Kramer

**Affiliations:** ^1^ Department of Cardiothoracic and Vascular Surgery, Deutsches Herzzentrum der Charité (DHZC), Berlin, Germany; ^2^ Charité—Universitätsmedizin Berlin, Corporate Member of Freie Universität Berlin and Humboldt-Universität zu Berlin, Berlin, Germany; ^3^ Department of Congenital Heart Disease—Pediatric Cardiology, Deutsches Herzzentrum der Charité (DHZC), Berlin, Germany; ^4^ Department of Congenital and Pediatric Heart Surgery, Deutsches Herzzentrum der Charité (DHZC), Berlin, Germany; ^5^ Partner Site Berlin, DZHK (German Center for Cardiovascular Research), Berlin, Germany; ^6^ Translational Cardiovascular Technologies, Institute of Translational Medicine, Department of Health Sciences and Technology, Swiss Federal Institute of Technology (ETH) Zurich, Switzerland; ^7^ Steinbeis Hochschule, Steinbeis-Transfer-Institut Kardiotechnik, Berlin, Germany

**Keywords:** transvenous lead extraction, rotational extraction sheaths, congenital heart disease, cardiac implantable electronic device, children

## Abstract

**Objectives:**

Due to the limited longevity of endovascular leads, children require thoughtful lifetime lead management strategies including conservation of access vessel patency. Consequently, there is an increasing interest in transvenous lead extraction (TLE) in children, however, data on TLE and the use of powered mechanical dissection sheaths is limited.

**Methods:**

We performed a retrospective cohort study analyzing all children <18 years that underwent TLE in our institution from 2015 to 2022. Procedural complexity, results and complications were defined as recommended by recent consensus statements.

**Results:**

Twenty-eight children [median age 12.8 (interquartile range 11.3–14.6) years] were included. Forty-one leads were extracted [median dwell time 85 (interquartile range 52–102) months]. Extractions of 31 leads (76%) in 22 patients (79%) were complex, requiring advanced extraction tools including powered bidirectional rotational dissection sheaths in 14 children. There were no major complications. Complete procedural success was achieved in 18 (64%) and clinical success in 27 patients (96%), respectively. Procedural success and complexity varied between lead types. The Medtronic SelectSecure™ lead was associated with increased odds of extraction by simple traction (*p* = 0.006) and complete procedural success (*p* < 0.001) while the Boston Scientific Fineline™ II lead family had increased odds of partial procedural failure (*p* = 0.017).

**Conclusions:**

TLE with the use of mechanical powered rotational dissection sheaths is feasible and safe in pediatric patients. In light of rare complications and excellent overall clinical success, TLE should be considered an important cornerstone in lifetime lead management in children. Particular lead types might be more challenging and less successful to extract.

## 1. Introduction

Children with congenital dysrhythmias, channelopathies or congenital heart disease (CHD) frequently require implantation of a pacemaker (PM) or implantable cardioverter defibrillator (ICD) in an early stage of life. In smaller children, cardiac implantable electronic devices (CIED) with epicardial leads are often used, due to the limited possibilities of transvenous lead access and the risk for thrombotic vascular complications given the comparably large diameter of lead bodies (1, 2). While short-term performance is comparable to transvenous leads, epicardial leads may have inferior long-time performance, especially in patients with CHD, and lead replacement will require thoracotomy (3–6). Therefore, primary implantation of transvenous leads or conversion to transvenous leads in case of epicardial lead malfunction is generally preferred in older children (2, 3). However, due to somatic growth and higher levels of physical activity, among other causes, resulting in increased mechanical strain, failure of transvenous leads is frequent in children (7). CIED and lead revisions may also be indicated due to system infection, system upgrade and lead recall or advisory status. There is a large experience with lead extractions in adult patients with CIED and various techniques and extractions tools were developed over recent decades (8–11). Indications for lead extractions for adult patients have been recommended by current consensus statements (12, 13). In children, however, data on transvenous lead extractions is limited to a small number of observational studies (14–20). Accordingly, due to the limited evidence, current consensus statements for CIED therapy in children have largely adopted recommendations on lead extractions from the experience in adult patients but emphasize important knowledge gaps (21). Comparable to adults, the use of various advanced extraction tools has been reported including radiofrequency, laser and mechanical powered sheaths (14, 16, 17, 18, 22). However, in particular data on the use of new generation mechanical rotational sheaths is limited to small numbers of pediatric patients (17, 23, 24).

Clinical practice concerning the decision of extracting or abandoning malfunctioning transvenous leads in children is variable and long-term prospective studies on lead abandonment vs. extraction are missing (14, 15, 25). However, as children dependent on CIED therapy will require functional leads for decades to come, lifetime lead management strategies and conservation of access vessel patency are mandatory. Procedural complexity of lead extraction and the need for advanced extraction techniques are associated with increasing lead age among other factors (16, 19). The incidence of major complications of lead extractions in children is fortunately very low with reported rates of 0%–4% and exceptionally rare procedure related mortality (14–19). With these considerations, it has become our institutional policy to attempt transvenous lead extraction in children whenever lead revision is required.

In light of the limited available data, the aim of our present study was to investigate our institutional results with lead extraction procedures in children.

## 2. Material and methods

**Figure 1:**
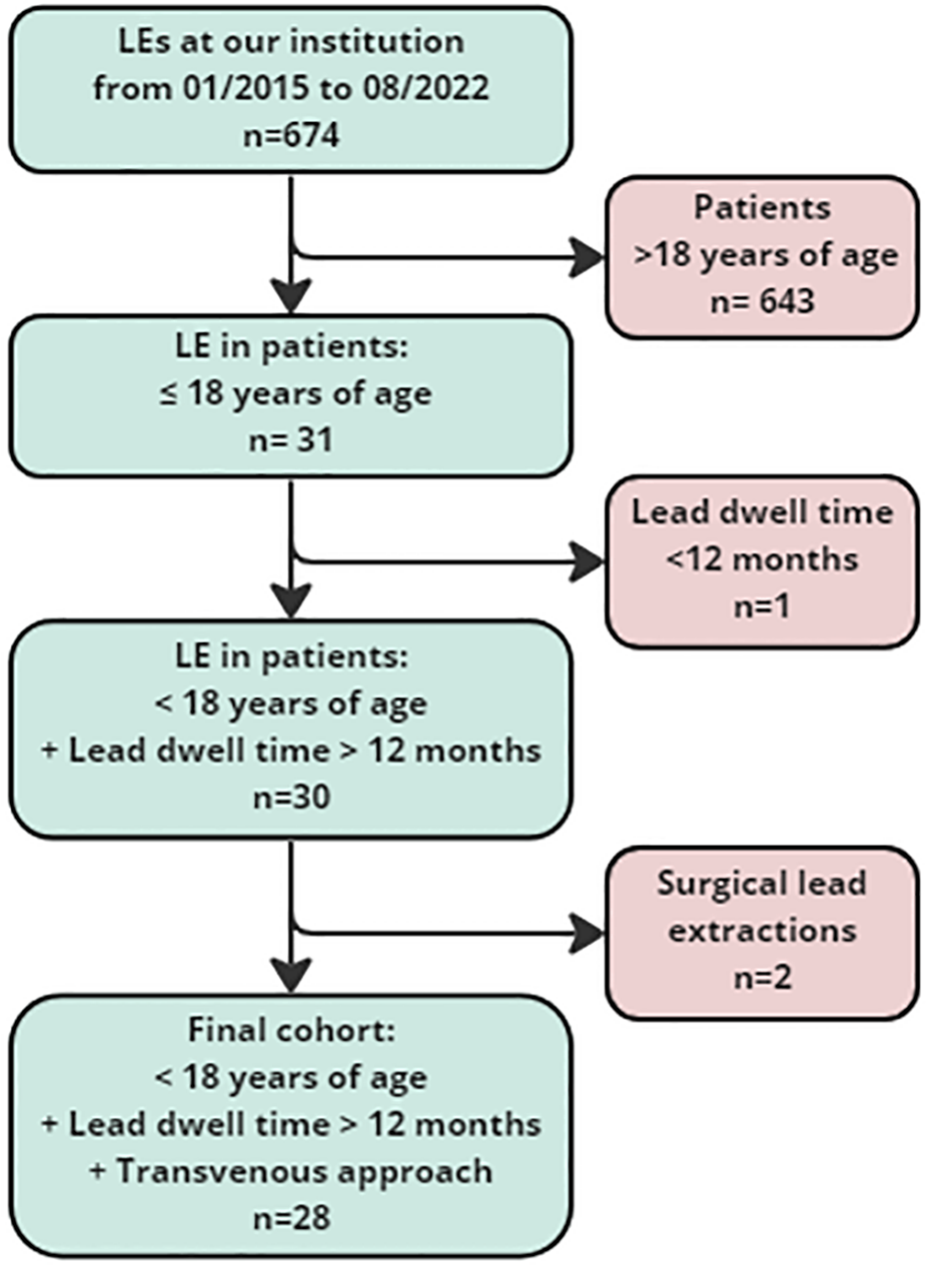
Study cohort. LE, Lead extraction.

We performed a retrospective cohort study reviewing patients that underwent transvenous lead extraction (TLE) in our institution during the study period 2015–2022. Patients were included in the study if they were <18 years of age at the time of TLE. Exclusion criteria were lead dwell time <12 months and surgical lead extraction. If patients underwent more than one extraction procedure during the study period, only the most recent was analyzed. The development of the final cohort is displayed in Figure 1. Demographic, clinical, CIED related and TLE procedural data were retrieved from patients' electronic charts and analyzed. The study was approved by the institutional review board and ethics committee (EA2/278/20), individual informed consent was not required due to the retrospective nature of this study.

### 2.1. Definitions

Procedural results were defined according to the definitions of the 2017 Heart Rhythm Society and the 2018 European Heart Rhythm Association expert consensus (12, 13). Complete procedural success for the extraction of an individual lead was defined as the removal of all components of that lead from the vascular space without the occurrence of a fatal or permanently disabling complication. A procedure was considered a clinical success if it attained the intended clinical outcome and did not involve the retention of any lead portion >4 cm in length. Complications were defined as undesired occurrences related to the procedure. They were classified as minor complications if they required medical intervention or minor procedural intervention or as major complications if they were life-threatening, resulted in death or caused significant or persistent disability, prolongation of the hospital admission or required additional significant therapeutic intervention such as cardiac surgery, pericardiocentesis, or vascular surgery (12, 13). Extraction procedures were classified as simple if only manual traction with non-locking or locking stylets was used or as complex, if advanced extraction tools were required, respectively (14, 16, 19).

### 2.2. Transvenous lead extraction

All procedures were performed by pediatric cardiologists experienced in CIED therapy and TLE according to current consensus statements recommendations (12). TLE was performed in a hybrid operating suite with monoplane fluoroscopic guidance. Patients were intubated and managed under general anesthesia. A radial arterial line as well as a jugular central venous line were placed and the patient's chest was prepped for emergency sternotomy. Additionally, arterial and venous femoral vascular access was established to allow emergency femoral cardiopulmonary bypass cannulation as well as to permit temporary transvenous pacing and access for a possible femoral extraction approach or possible interventions such as emergency vessel occlusion or lead access vessel revascularization. Transesophageal echocardiography was in place throughout the procedure to allow intraprocedural monitoring and exclusion of cardiovascular complications due to TLE. During TLE procedure, a cardiac surgical team as well as a perfusionist were on standby until leads were extracted and cardiovascular complications ruled out. For lead extraction, a multistep approach was applied. In general, TLE was performed primarily via the lead access vessel. Initially, simple manual traction with a non-locking stylet inserted and, if possible, retraction of the active fixation helix was attempted. If unsuccessful, a locking stylet and compression coil (Liberator®, One-tie®, Cook Medical, USA) were used to support manual traction. In case manual traction was insufficient for lead extraction, advanced extraction tools were employed such as polypropylene telescope dissection sheaths (Byrd Dilator Sheath, Cook Medical, USA) or mechanical powered rotational dissection sheaths (Evolution® RL, Cook Medical, USA). Lumenless leads were extended (Bulldog™ lead extender, Cook Medical, USA). If primary complete extraction of leads failed, extraction of remaining lead components via femoral access was attempted using snare catheters (Needle's Eye Snare®, Cook Medical, USA; EN Snare®, Merit Medical Systems, USA; Multi-Snare®, PFM Medical, Germany).

### 2.3. Outcomes

Primary outcome was procedural success; secondary outcomes were complexity of extraction procedures and complications.

### 2.4. Statistics

Statistical analyses were performed using SPSS statistics software (version 25, IBM Corp., NY, USA). Data distribution was tested using D'Agostino-Pearson test. Variables are expressed as figures (percentages) and median [interquartile range, IQR]. Continuous variables were compared using non-parametric Mann–Whitney test. Chi–Square-Test was used for comparison of categorical data. Potential factors associated with primary or secondary outcome were evaluated with univariable logistic regression analysis. *P*-values <0.05 were considered statistically significant.

## 3. Results

### 3.1. Patient cohort

Patient data are provided in Table 1. Twenty-eight pediatric patients who underwent TLE were included in the study (Figure 1). The median age at the first transvenous lead implant was 4.3 [IQR 2.3–7.9] and 6.2 [IQR 3.1–8.5] years at the implantation of indwelling leads, respectively. The median age at TLE was 12.8 [IQR 11.3–14.6] years. Ten patients (35%) had CHD, 8 patients (29%) had a channelopathy and 7 patients (25%) had a congenital dysrhythmia. The remaining patients had acquired dysrhythmias of unknown or presumably inherited origin (*n* = 4, 14%) and hypertrophic cardiomyopathy with recurrent ventricular tachycardia (*n* = 1, 4%). Two patients had more than one diagnosis. The most frequent indications for CIED treatment included complete AV block (*n* = 17, 61%), long QT syndrome (*n* = 6, 21%), sinus node dysfunction (*n* = 6, 21%) and ventricular tachycardia (*n* = 3, 11%), several patients had more than one indication.

**Table 1:** Patient characteristics.

Patients characteristics	*n* = 28
Male (*n*)	19 (68%)
Weight (kg)	47.3 [37.1–54.5]
Height (cm)	159 [146–165]
Body surface area (m²)	1.46 [1.22–1.59]
Age at previous lead implantation (years)	6.2 [3.1–8.5]
Age at first transvenous lead implantation (years)	4.3 [2.3–7.9]
Age at TLE (years)	12.8 [11.3–14.6]
CIED implantation site: right/left (*n*)	3 (11%)/25 (89%)
Diagnoses^a^	
CHD (*n*)	10 (36%)
Channelopathy (*n*)	8 (29%)
Congenital dysrhythmia (*n*)	7 (25%)
Acquired dysrhythmia (*n*)	4 (14%)
HCM (*n*)	1 (4%)
Indications for CIED^a^	
Complete AVB	17 (61%)
Sinus node dysfunction	6 (21%)
Long-QT syndrome	6 (21%)
VT primary/secondary prophylaxis	3 (11%)
Indications for TLE^a^	
Infection (*n*)	1 (4%)
Lead malfunction (*n*)	17 (61%)
Growth related lead distortion (*n*)	5 (18%)
CIED upgrade (*n*)	4 (14%)
Abandoned leads (*n*)	4 (14%)
VT triggered by prolapsed lead loop (*n*)	1 (4%)

AVB, atrioventricular block; CIED, cardiac implantable electronic device; CHD, congenital heart disease; HCM, hypertrophic cardiomyopathy; TLE, transvenous lead extraction; VT, ventricular tachycardia.

Data is reported as frequency (percentage) or median [interquartile range].

^a^ Note that several patients had multiple diagnoses and indications.

### 3.2. Procedural details

**Figure 2:**
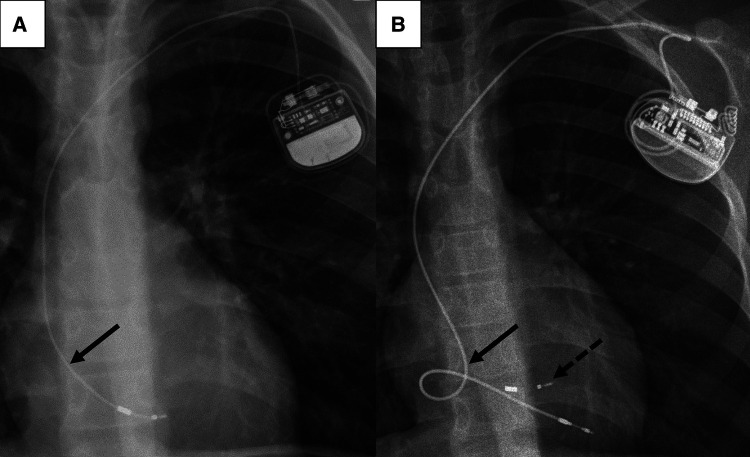
Chest radiographs of a 12-year-old boy before (**A**) and after (**B**) TLE. (**A**): depleted length reserve (arrow) of the right ventricular lead due to somatic growth. (**B**): lead length reserve for anticipated further somatic growth of the new lead implanted after TLE. The lead “reserve loop” is positioned in the right atrium (arrow). A fragment of the lead tip from the previously implanted 4472 model (Boston Scientific Fineline™ II) lead (dotted arrow) remained in the right ventricle. TLE, transvenous lead extraction.

In the 28 included patients, a total of 41 leads were extracted with a median dwell time of 85 [IQR 52–102] months (Table 2). The most frequent primary indications for lead revision were malfunction (*n* = 17, 61%) and somatic growth related lead distortion (*n* = 5, 18%) while CIED infection was rare (*n* = 1, 4%). Growth related lead distortion as presumable cause was also present in the majority of patients with lead malfunction (13/17, 76%) (Figure 2). In those patients with distorted leads without malfunction, advancing present leads within access vessels [*n* = 8 leads, median dwell time of 88 (IQR 51–120) months] was primarily intended but unsuccessful in all cases due to vascular adhesions.

**Table 2:** Characteristics of extracted leads.

Lead characteristics		*n* = 41
Lead dwell time (months)		85 [52–102]
High voltage leads (*n*)		4 (10%)
Single Coil (*n*)		4 (100% of high voltage leads)
Active fixation (*n*)		40 (98%)
Right atrial leads (*n*)		14 (34%)
Right ventricular/sub-pulmonary ventricular leads (*n*)		27 (66%)
Lead models (Manufacturer, model)		
Boston Scientific	0292	2 (5%)
	0272	1 (2%)
	4471	1 (2%)
	4472	8 (20%)
	4473	10 (24%)
	4474	2 (5%)
Medtronic	3830	10 (24%)
	5076	1 (2%)
	5594	1 (2%)
St. Jude/Abbott	7122	1 (2%)
	2088	1 (2%)
Biotronik	Unknown	2 (5%)
Osypka	K5Y	1 (2%)

Data is reported as frequency (percentage) or median [interquartile range].

Median duration of all TLE procedures (skin-to-skin time) was 162 [IQR 118–200] minutes, median fluoroscopy time was 12.8 [IQR 6.7–22.3] minutes. New leads and, if indicated, generators were implanted in all but two patients during TLE procedures. One patient with hypertrophic cardiomyopathy underwent bi-ventricular assist device implantation due to terminal heart failure. Explantation of her single chamber ICD and lead extraction prior to pending cardiac transplantation was favored and TLE was performed concomitantly with assist device implantation. The second patient had CIED infection. His single chamber pacemaker had been implanted due to intermittent sinus node dysfunction, however, he had low rates of atrial pacing and was therefore judged to be stable enough without temporary pacing. A new pacemaker was implanted subsequently 8 days later.

Extractions of 31 leads (76%) in 22 patients (79%) were considered complex, requiring advanced extraction tools. In 14/22 patients (64%), powered bidirectional rotational dissection sheaths (Cook Evolution® RL and/or Evolution® Shortie RL) were used. Snare catheters to retrieve lead remnants via femoral approach were used in 8 of these 22 patients (36%) in 11 leads. Extraction characteristics are provided in Table 3.

**Table 3:** Procedural details.

Parameter	
Duration (min)	162 [118–200]
Fluoroscopy time (min)	12.8 [6.7–22.3]
Dose area product (mGy/cm²)	4,647 [2,329–7,170]
Complex extraction (*n*)	31/41 leads (76%) 22/28 patients (79%)
Extraction tools	*n* = 41 leads
Locking stylet (*n*)	31 (76%)
Any mechanical powered sheath (*n*)	19 (46%) 14/28 patients (50%)
Evolution® Shortie, RL 9 F and/or 11 Fr (*n*)	9 (22%)
Evolution® RL, 9F, 11F and/or 13F (*n*)	17 (41%)
Telescope dilator sheaths 7–10F (*n*)	12 (29%)
Snare catheter (*n*)	11 (27%)

Data is reported as frequency (percentage) or median [interquartile range]. Procedure duration (skin-to-skin time), fluoroscopy time and dose area product are reported including implantation of a new cardiac implantable electronic device system.

### 3.3. Procedural success

There were no major complications. Tricuspid valve/subpulmonary atrioventricular valve function was unchanged in all patients as documented by transesophageal echocardiography. All patients survived to hospital discharge, median length of hospital stay was 4 [IQR 3–6] days. Minor complications occurred in three patients (11%). One experienced an episode of bradycardia during TLE, which was managed medically without further consequences. The second patient developed episodes of non-sustained ventricular tachycardia during the attempt of extracting remaining fragments of the extracted ventricular lead with a snare catheter. The attempts were abandoned and the dysrhythmias resolved without further interventions required. In a third patient, small lead fragments dislodged to a hepatic vein and could not be retrieved. This, however, was without clinical consequences.

Outcome characteristics are summarized in Table 4. Complete procedural success was achieved in 18 patients (64%) while clinical success was achieved in almost all patients (*n* = 27, 96%). Partial procedural success with retention of small lead fragments <4 cm were in particular observed with the Boston Scientific Fineline lead models 447x (4471, 4472, 4473, 4474) in our cohort. Frequently, these lead types fragmented just at the tip during extraction, and the lead tips could not be retrieved (Figure 2). The other lead type with only partial procedural success was a bipolar Osypka K5Y lead, which disintegrated at the tip during extraction and small fragments of the lead tip embolized to a hepatic vein. The additional use of femoral access for the extraction of lead fragments with snare catheters was significantly more often required with the 447x lead types compared to other lead types (10/21 vs. 1/20, Chi² *p* = 0.002).

**Table 4:** Outcome characteristics.

Outcome variable	*n* = 28 patients
Minor Complications (*n*)	3 (11%)
Major Complications (*n*)	0 (0%)
Procedure mortality (*n*)	0 (0%)
30-day mortality	0 (0%)
Length of hospital stay (days)	4 [3–6]
Complete procedural success (*n*)	18/28 patients (64%) 31/41 leads (76%)
Clinical Success (*n*)	40/41 leads (98%) 27/28 patients (97%)

Data is reported as frequency (percentage) or median [interquartile range].

TLE, transvenous lead extraction.

Angiography prior to TLE revealed thrombotic obstructions of access vessels, mostly of the innominate vein, in a considerable number of patients (partial *n* = 6, 21%, complete *n* = 2, 7%). In all but one patient, access vessel patency was maintained or achieved by TLE. Two patients (7%), however, required additional balloon angioplasty due to stenosis of the innominate vein after recanalization of complete (*n* = 1) or partial (*n* = 1) thrombotic obstruction. In one patient during extraction with a powered rotational dissection sheath, the leads fragmented at the level of the completely obstructed innominate vein. Leads could be successfully extracted via femoral approach by snare catheters; however, recanalization of the innominate vein was unsuccessful.

### 3.4. Factors associated with procedural success and procedural complexity

Analysis by type of the two most frequently used leads in our cohort shows that the 3830 model was significantly associated with complete procedural success (Chi² *p* = 0.039, univariable regression *p* < 0.001). In contrast, the 447x models were significantly associated with decreased odds of complete success (*p* = 0.017). None of the additional parameters analyzed was associated with procedural success (Table 5).

**Table 5:** Univariable analysis of predictors for procedural success and complex extraction.

Procedural success (analysis per lead)					
Variable	Procedural Success (*n*)	Partial failure (*n*)	OR	95% CI	*P*-value
Age at extraction (years)	31	10	1.056	0.791–1.411	0.710
Age at extraction ≥13 years (*n*)	21/31	4/10	3.150	0.723–13.732	0.127
Age at lead implantation (years)	31	10	1.206	0.933–1.560	0.153
Age at lead implantation ≥7 years (*n*)	17/31	3/10	2.833	0.616–13.037	0.181
Lead dwell time (years)	31	10	0.988	0.986–1.008	0.245
Lead dwell time ≥7 years (*n*)	14/31	7/10	0.353	0.077–1.624	0.181
CHD (*n*)	13/31	3/10	1.685	0.365–7.776	0.504
Previous transvenous extraction (*n*)	12/31	1/10	5.684	0.637–50.726	0.120
Lead model 447x vs. other (*n*)	12/31	9/10	0.070	0.008–0.626	**0**.**017**
Lead model 3830 vs. other (*n*)	10/31	0/10	N/A	N/A	**<0**.**001**
Complex extraction (analysis per lead)					
Variable	Simple traction (*n*)	Complex extraction (*n*)	OR	95% CI	*P*-value
Age at extraction (years)	10	31	1.343	1.018–1.771	**0**.**037**
Age at extraction ≥13 years (*n*)	5/10	20/31	1.818	0.430–7.685	0.416
Age at lead implantation (years)	10	31	1.280	1.032–1.587	**0**.**024**
Age at lead implantation ≥7 years (*n*)	2/10	18/31	5.538	1.006–30.494	**0**.**049**
Lead dwell time (years)	10	31	0.997	0.977–1.017	0.761
Lead dwell time ≥7 years (*n*)	6/10	15/31	0.625	0.147–2.659	0.525
CHD (*n*)	5/10	11/31	0.550	0.130–2.325	0.416
Previous transvenous extraction (*n*)	1/10	12/31	5.684	0.637–50.726	0.120
Lead model 447x vs. other (*n*)	3/10	18/31	3.231	0.700–14.900	0.133
Lead model 3830 vs. other (*n*)	6/10	4/31	0.099	0.019–0.511	**0**.**006**

Univariable logistic regression analysis of predictors for primary and secondary outcomes procedural success vs. partial procedural failure and complex extraction vs. simple manual traction. Analysis was performed on per lead basis. Age at lead extraction, age at previous lead implant and lead dwell time were analyzed as continuous variables as well as dichotomized at median values. *P*-values <0.05 indicating statistical significance are given in bold. 447x refers to the Boston Scientific Fineline™ lead models encountered in the study cohort 4472, 4473 and 4474.

CI, confidence interval; CHD, congenital heart disease; N/A, not applicable; OR, odds ratio.

While median dwell times did not differ significantly between the 3830 model leads and other leads (72 [IQR 49–95] vs. 85 [IQR 54–111] months, *p* = 0.501), TLE by simple manual traction was significantly more frequent in the 3830 lead model (Chi² *p* = 0.003, univariable regression *p* = 0.006). In addition, older age at TLE was significantly associated with increased odds of complex extraction while additionally analyzed parameters did not show a significant association (Table 5).

## 4. Discussion

In our study summarizing our institutional experience with TLE in children, we demonstrate that TLE can be successfully and safely performed in the pediatric population. There were no major complications or mortalities despite the high rate of complex extractions and the frequent use of powered rotational dissection sheaths in our cohort. Clinical success was achieved in 97% of patients. This compares well with the results from previous studies with clinical success rates of 80%–98% in children or mixed cohorts and approximately 98% in large registries of adult patients reported in recent years (8, 11, 14–17).

The numbers of children and young adults with CIED therapy is increasing (26, 27). Children in particular have a limited durability of transvenous PM and ICD leads (3, 4, 7, 28). One reason for that is somatic growth related lead distortion and subsequent malfunction, which is frequently observed in children even though lead reserve loops, anticipating growth, are generally placed during CIED implantations (Figure 2) (7, 29). However, children with CIED therapy are dependent on functional leads for decades of life expectancy. Consequently, life time lead management strategies are mandatory and conservation of access vessel patency is paramount in particular. In fact, 8 of our patients (29%) had partial or complete thrombotic obstruction of the subclavian and/or innominate vein prior to TLE. While some authors have described uncomplicated short-term outcomes of abandonment of malfunctioning leads in young patients, long-term prospective studies are missing (25). Abandoning superfluent leads, in particular in children with smaller vessel dimensions, might impede later vascular access due to thrombotic venous occlusion (15, 30). Moreover, lead abandonment may result in interactions with other leads, cause regurgitation of the tricuspid valve/subpulmonary atrioventricular valve and increase the risk of infection (31). Also, abandoned leads may pose a contraindication for magnetic resonance imaging, although these concerns might require reconsideration in the light of more recent clinical data (32, 33).

In apprehension of life time lead management, there has been an increasing interest in TLE in children. However, in contrast to adult patients, the overall data on TLE in children is restricted to a small number of single institutional studies with limited cohort sizes or mixed pediatric/adult populations (14–20, 22). TLE procedures in children are often complex and require advanced extraction tools in approximately 30%–70% (14, 16, 17). Accordingly, the use of powered sheaths, including radiofrequency, laser and mechanical powered sheaths, has previously been reported with good success in pediatric patients (14, 16, 17, 18, 22). However, in particular data on the use of new generation mechanical rotational sheaths, such as the Evolution® RL (Cook Medical) is limited to very small numbers of pediatric patients (17, 23, 24). One study with a larger, mixed cohort of children and young adults reports the use of mechanical powered sheaths in 48/118 complex TLE, however the study did not specify in which age group mechanical sheaths were used (16). In our cohort, the Evolution® RL (sizes from 9–13F) mechanical rotational dissection sheath was used in 14 pediatric patients without complications. Our youngest patient in whom a mechanical powered sheath was used was 10 years of age (weight 31.8 kg); however, a previous case report describes the use in a child as young as 7 years (weight 17 kg) (24). In 7/8 patients with thrombotic obstruction of access vessels prior to TLE, patency was achieved successfully by TLE; two of these patients required balloon angioplasty while no patient required stent implantation. Our limited data suggests that the Evolution® RL can effectively and safely be used for TLE in children. Thrombotic occlusion of access vessel may be successfully recanalized by the use of powered mechanical sheaths.

Interestingly, in our study we demonstrate differences in extraction complexity and procedural success according to lead types. The 3830 model (Medtronic SelectSecure™) showed a decreased odd for complex TLE. After a median dwell time of 72 [IQR 49–95] months, 6/10 leads could be extracted by manual traction and all leads could be extracted with complete procedural success. The 3830 model is a polyurethane insulated lead that differs from other lead models by its low diameter (4.1F) and lumenless isodiametric design with a central cable, probably offering more tensile strength. Due to its small lead body dimensions it is frequently used in the pediatric population. A good extractability of the 3830 lead with a lower number of complex extractions and a higher procedural success rate has also previously been observed in cohorts of children and young adults as well as pediatric and adult CHD patients (16, 34). The 4471–4474 lead models (Boston Scientific Fineline™ II) are also small diameter leads (5–6F) often used in children with a coaxial design. In our cohort, we observed an increased incidence of partial procedural failure with the 447x lead family. In 9/21 leads, small fragments <4 cm length remained that could not be extracted. Typically, the lead fractured at the tip (Figure 2) Our findings are in line with a recent study that also reported a high rate of lead fracture during TLE in the Fineline™ family (35). Moreover, extraction of these lead model types might more frequently require complex extraction techniques (12). This was also observed in our cohort. A femoral approach with additional snaring of lead fragments was significantly more frequently required with Fineline™ leads.

### 4.1. Limitations

Limitations of this study are inherent to its retrospective design and restriction to a single institution. Importantly, the number of patients in our study is small, limiting the options of statistical analyses such as multivariable analyses and therefore the possibilities of confident conclusions. Most extractions in children were performed by two extractors (BP, PK) and preferences for approaches may have varied. Other frequently used advanced extractions techniques such as laser powered sheaths were not employed in our study. Due to our good institutional experience with mechanical powered sheaths for TLE and with more recent data suggesting higher complications rates and lower success rates with laser powered sheaths, this technique was not adopted in our institution (36). A considerable number of patients were referred to our center by other institutions, thus previous implantation techniques and choice of lead types varied, which might have influenced the procedural success of lead extractions.

## 5. Conclusions

TLE can be safely and effectively performed in children. A large number of procedures require complex extraction techniques which emphasizes the requirement of adequate experience and resources for TLE in children. The use of advanced extraction tools such as the Evolution® RL mechanical powered rotational dissection sheath is feasible and safe in the pediatric population. While overall clinical success of TLE is excellent, extraction of particular lead types such as the Boston Scientific Fineline™ lead family might be more difficult and frequently result in only partial procedural success. A considerable number of thrombotic venous obstructions observed in children with transvenous CIED leads suggests that lead abandonment might not be an adequate strategy in light of the requirement of life-long CIED therapy. Larger studies and preferably multi-institutional prospective registries are required to reveal patient-, lead- and technique-related aspects that might have impact on the success and complexity of TLE in children.

## Data Availability

The data that support the findings of this study are available on request from the corresponding author in accordance with applying data protection legislation. The data are not publicly available due to privacy or ethical restrictions.
